# Effect of dietary resveratrol on the metabolic profile of nutrients in obese OLETF rats

**DOI:** 10.1186/1476-511X-12-8

**Published:** 2013-02-04

**Authors:** Koji Nagao, Tomoyuki Jinnouchi, Shunichi Kai, Teruyoshi Yanagita

**Affiliations:** 1 Department of Applied Biochemistry and Food Science, Saga University, Saga, 840-8502, Japan; 2 Department of Health and Nutrition Sciences, Nishikyushu University, Kanzaki, 842-8585, Japan

**Keywords:** Resveratrol, Respiratory gas analysis, Metabolic profile, Obesity, OLETF rat

## Abstract

**Background:**

Resveratrol (*trans*-3,4^′^,5-trihydroxystilbene) is a naturally occurring phytoalexin produced by plants in response to various stresses. Several studies have shown that resveratrol is present in significant amounts in a variety of human diets, including wines, grapes, berries, and peanuts, and it possesses several beneficial health properties, such as atheroprotective, anti-obesity, anti-cancer, anti-inflammatory and antioxidant activities. In this study, we evaluated the effect of resveratrol on the pathogenesis of obesity and the metabolic profile of nutrients in non-high fat-fed obese OLETF rats.

**Results:**

Although lipid parameters in the serum and liver were not changed, the accumulation of abdominal white adipose tissues was markedly prevented in resveratrol diet-fed OLETF rats after 4 weeks of feeding. The results of the respiratory gas analysis indicated that dietary resveratrol induced the partial enhancement of fat metabolism and sparing actions for carbohydrate and protein at 1 week and 3 weeks of feeding in OLETF rats. Additionally, the adipose mRNA level of carnitine palmitoyltransferase in the resveratrol diet-fed OLETF rats was higher than the control rats after 4 weeks of feeding.

**Conclusion:**

Our study demonstrated that dietary resveratrol can prevent obesity through a change in the metabolic profile of nutrients in obese OLETF rats.

## Background

Lifestyle-related diseases, such as hyperlipidemia, arteriosclerosis, diabetes mellitus, and hypertension, are widespread and increasingly prevalent in industrialized countries and contribute to the increase in cardiovascular morbidity and mortality [1,2]. Accompanied by the rapid increase in the number of elderly people, they are important not only medically but also socioeconomically. Although the pathogenesis of lifestyle-related diseases is complicated and the precise mechanisms have not been elucidated, obesity has emerged as one of the major cardiovascular risk factors according to epidemiologic studies [3-5]. Obesity is defined as an increased mass of adipose tissue, and its prevalence and severity are markedly increasing in Westernized countries.

Diet has been recognized as a factor that contributes to the development and prevention of lifestyle-related diseases, and various natural molecules in plants and fruits have been used in folk medicines throughout the world for treating those diseases [6-10]. Resveratrol (*trans*-3,4^′^,5-trihydroxystilbene) is a naturally occurring phytoalexin produced by plants in response to various stresses. Several studies have shown that resveratrol is present in significant amounts in a variety of human diets, including wines, grapes, berries, and peanuts, and it possesses several beneficial health properties, such as atheroprotective, anti-cancer, anti-inflammatory and antioxidant activities [11-13]. Recent studies have also reported that resveratrol has anti-obesity effects in high-fat-fed mice and rats [14,15]. However, the effects of resveratrol on the metabolic profile of nutrients and body fat accumulation in non-high-fat diet-fed rodents have not been fully evaluated. Otsuka Long-Evans Tokushima fatty (OLETF) rats develop a syndrome with multiple metabolic and hormonal disorders that shares many features with human obesity [16-18]. OLETF rats have hyperphagia because they lack receptors for cholecystokinin, and they become obese even by consuming a normal diet (AIN-76, 7% fat diet) [19-21]. Therefore, we evaluated the effect of resveratrol on the pathogenesis of obesity and the metabolism of carbohydrate, fat, and protein in obese OLETF rats.

## Materials and methods

### Animals and diets

All aspects of the experiment were conducted according to the guidelines provided by the ethical committee for experimental animal care of Saga University. Six-week-old male OLETF rats were purchased from Hoshino Laboratory Animals, Inc. (Ibaraki, Japan). The rats were individually housed in metal cages in a temperature-controlled room (24°C) under a 12 h light/dark cycle. After a 1-week adaptation period on a powder chow diet (CE-2, Clea Japan, Tokyo), they were assigned to two groups (six rats each), each fed one of two diets: (i) a semi-synthetic diet containing (in weight %): casein, 20; corn oil, 7; cornstarch, 15; vitamin mixture (AIN-76™), 1; mineral mixture (AIN-76™), 3.5; DL-methionine, 0.3; choline bitartrate, 0.2; cellulose, 5; and sucrose, 45 (control group); or (ii) a semi-synthetic diet supplemented with 0.5% of resveratrol (Wako Pure Chemicals, Tokyo, Japan) at the expense of sucrose. The animals received the different diets for 4 weeks. The composition of the semi-synthetic diets is shown in Table 1. The rats were subjected to respiratory gas analysis after 1 week and 3 weeks of consuming the experimental diets. At the end of the feeding period and after a 9 h starvation period, all rats were killed by aortic exsanguination under diethyl ether anesthesia. The abdominal (perirenal, epididymal, and omental) white adipose tissue (WAT) and livers were excised immediately, and the serum was separated from the blood.

**Table 1:** The composition of the experimental diets

**Ingredients**	**Control**	**Resveratrol**
	%	
Casein	20.0	20.0
Corn starch	15.0	15.0
Cellulose	5.0	5.0
Mineral mixture (AIN-76)	3.5	3.5
Vitamin mixture (AIN-76)	1.0	1.0
DL-Methionine	0.3	0.3
Choline bitartrate	0.2	0.2
Corn oil	7.0	7.0
Resveratrol		0.5
Sucrose	48.0	47.5

### Measurement of the triglyceride, cholesterol and glycogen levels in the liver

The liver lipids were extracted, and the concentrations of triglyceride and cholesterol were determined as described elsewhere [22]. The glycogen levels were determined according to the method of Lo et al. [23].

### Measurement of the serum parameters

The serum triglyceride, cholesterol, and glucose levels were measured using commercial enzyme assay kits (Wako Pure Chemicals, Tokyo, Japan).

### Respiratory gas analysis

The instruments and software used for the measurement of the oxygen consumption and respiratory quotient in the rats were obtained from Arco system (Chiba, Japan). The system consisted of six acrylic metabolic chambers, a mass spectrometer (model WSMR-1400), a gas sampler (model WGSS-1000), and a switching controller (model WMSC-2000). Each metabolic chamber had a 210 cm^2^ floor and was 11.5 cm in height. Room air was pumped through the chambers at a rate 1.8 L/min. Expired air was dried in a cotton-thin column and then directed to a mass spectrometer. Air from each chamber was sampled for 1 min. Therefore, air from each chamber was measured every 7 min, and data were recorded in a spreadsheet. The respiratory quotient (RQ) and energy production rate were calculated by the software using the following formulas: RQ = VCO_2_/VO_2_ and energy production rate = (2.96*RQ + 2.49)*VO_2_, where VO_2_ is the oxygen consumption, and VCO_2_ is the carbon dioxide exhaustion. Additionally, 24 h urine samples were collected, and oxygen production from protein metabolism (VO_2_-P) was calculated from the urinary nitrogen concentration (UN). The UN was determined using an assay kit (Urinary nitrogen test from Wako Pure Chemicals, Tokyo, Japan), and VO_2_-P was calculated using the following formula: VO_2_-P = UN*5.923. Thus, the energy metabolism was compared between the two groups using the values of non-protein oxygen consumption and non-protein energy expenditure. After 1 week or 3 weeks of consuming the semi-synthetic diets, each animal was placed into a metabolic chamber for 24 h respiratory gas analysis. During the analysis, the animals were pair-fed and had free access to water. The sampled gases were analyzed in a separate room to avoid stressing the rats.

### Analysis of mRNA expression

Total RNA was extracted from 0.1 g of WAT using an RNeasy Lipid Tissue Mini Kit (Qiagen, Tokyo, Japan). A TaqMan Universal PCR Master Mix (Applied Biosystems, Tokyo, Japan) and Assay-on-Demand Gene Expression Products (Rn00569117_m1 for fatty acid synthase (FAS), Rn00580702_m1 for carnitine palmitoyltransferase (CPT), Rn00566193_m1 for peroxisome proliferator-activated receptor (PPAR)-α, Rn00565707_m1 for PPAR-δ, Rn00440945_m1 for PPAR-γ, and Hs99999901_s1 for 18S RNA, Applied Biosystems) were used for quantitative real-time RT-PCR analysis of FAS, CPT, PPARα, PPARδ, PPARγ, and 18S RNA expression in the liver. Amplification was performed with a real-time PCR system (ABI Prism 7000 Sequence Detection System; Applied Biosystems). The results were quantified with a comparative method and expressed as a relative value after normalization to 18S RNA expression.

### Statistical analysis

All of the values are expressed as the mean ± standard error. The significance of the differences between the means of the two groups was determined by Student’s *t*-test. Differences were considered to be significant at *p* < 0.05.

## Results and discussion

### Effects of the resveratrol diet on growth parameters and lipid parameters in OLETF rats

**Figure 1:**
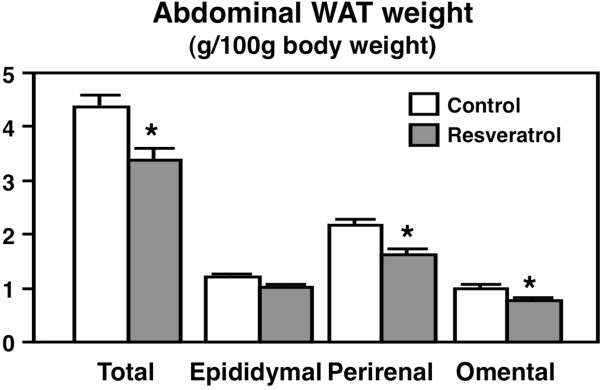
**The relative abdominal white adipose tissue weights in OLETF rats.** The rats were fed a control diet or the resveratrol diet for 4 weeks. The values are expressed as the mean ± standard error for six mice. See Table 1 for the composition of the diets. ^*^ Significant (*P* < 0.05) difference between the control and resveratrol groups of OLETF rats.

The two groups of OLETF rats did not differ in their initial body weight, final body weight, or food intake during or after the 4-week feeding period (Table 2). Although lipid parameters in the serum and liver were not changed (Table 2), the accumulation of abdominal WAT was markedly prevented in the resveratrol-diet-fed OLETF rats after 4 weeks of feeding (Figure 1). These results indicate that resveratrol can prevent obesity in non-high-fat diet-fed obese OLETF rats.

**Table 2:** The effect of resveratrol on growth and lipid parameters in OLETF rats

	**Control**	**Resveratrol**
Initial body weight (g)	132 ± 3	132 ± 3
Final body weight (g)	294 ± 3	299 ± 3
Food intake (g)	498 ± 3	499 ± 3
Serum cholesterol (mg/dL)	135 ± 4	122 ± 5
Serum triglyceride (mg/dL)	76.3 ± 5.0	96.5 ± 10.6
Hepatic cholesterol (mg/g liver)	3.34 ± 0.11	3.35 ± 0.13
Hepatic triglyceride (mg/g liver)	14.8 ± 1.4	16.4 ± 1.4

The values are expressed as the mean ± standard error for six mice.

### Effects of the resveratrol diet on nutrients oxidation in OLETF rats

**Figure 2:**
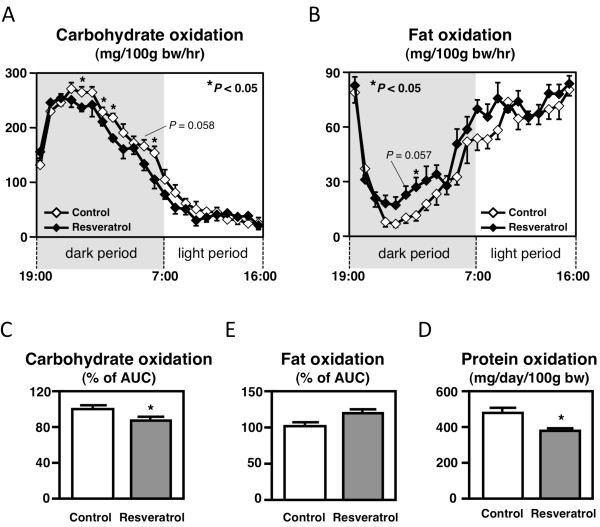
**Carbohydrate oxidation (A, C), fat oxidation (B, D), and protein oxidation (E) in the rats fed the control diet or the resveratrol diet at 3 weeks of feeding.** The values are expressed as the mean ± standard error for six mice. See Table 1 for the composition of the diets. ^*^ Significant (*P* < 0.05) difference between the control and resveratrol groups of OLETF rats.

Several studies have shown that functional food components produce anti-obesity effects by altering energy metabolism [24-27]. Their actions may have altered the energy expenditure or influenced the balance of nutrient metabolism, which affects body composition. To examine the effect of the resveratrol diet on the oxidation of nutrients, respiratory gas analysis was performed at 1 week and 3 weeks of feeding in OLETF rats. Because amount of food intake affects energy metabolism, rats were fed same amount of diets (12 g at 1 week and 14 g at 3 weeks) during the measurement. As the results, the total oxygen consumption and energy expenditure were not altered by dietary resveratrol at 1 week or 3 weeks of feeding (data not shown). However, carbohydrate oxidation (control, 4.87 ± 0.12; resveratrol, 4.43 ± 0.12 g/day/100 g body weight; *P* < 0.05) and protein oxidation (control, 0.402 ± 0.030; resveratrol, 0.286 ± 0.036 g/day/100 g body weight; *P* < 0.05) were significantly lowered in the resveratrol diet-fed OLETF rats at the 1 week of feeding period. Moreover, dietary resveratrol induced the partial enhancement of fat oxidation (Figure 2B) and suppressed carbohydrate (Figure 2A &2C) and protein (Figure 2E) oxidation after 3 weeks of feeding in OLETF rats. These results suggest that dietary resveratrol has an anti-obesity effect through the alteration of metabolic profiles of nutrients in obese OLETF rats.

### Effects of the resveratrol diet on the serum glucose level and hepatic glycogen content in OLETF rats

**Figure 3:**
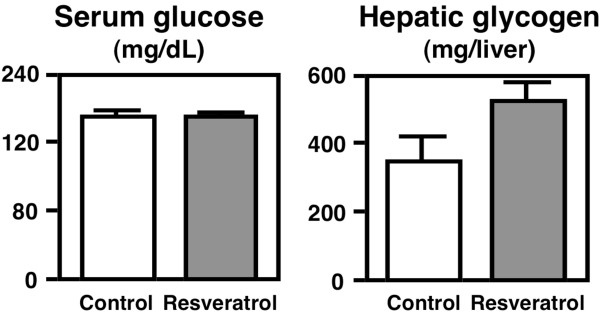
**Serum glucose levels and liver glycogen contents in OLETF rats.** The rats were fed a control diet or the resveratrol diet for 4 weeks. The values are expressed as the mean ± standard error for six mice. See Table 1 for the composition of the diets.

Glycogen is the storage form of carbohydrates and is primarily found in the liver and muscles, and hepatic glycogen is converted into glucose for use throughout the body. Fat (or adipose tissue) is also storage fuel and is recruited for energy fuel when glycogen stores are depleted. In addition, the depletion of glycogen stores has been associated with fatigue and converting structural proteins into fuel sources. Although serum glucose levels were not different between groups, the hepatic glycogen content was 51.6% (not statistically significant) higher in the resveratrol diet-fed OLETF rats after 4 weeks of feeding (Figure 3).

A growing amount of evidence has demonstrated that some strategies can make the body recruit fat rather than glycogen to meet energy demands [28]. A previous study demonstrated that AMPK activity and fuel selection in muscle in response to exercise can be manipulated by diet [29]. Another study also reported that AMPK activation in the liver leads to the stimulation of fatty acid oxidation and inhibition of lipogenesis, glucose production and protein synthesis [30]. Because dietary resveratrol has the potential to activate AMPK [13], it is possible that resveratrol induced sparing actions for carbohydrates and protein (which contribute to the anti-obesity effect) in OLETF rats.

### Effects of the resveratrol diet on adipose mRNA levels in OLETF rats

**Figure 4:**
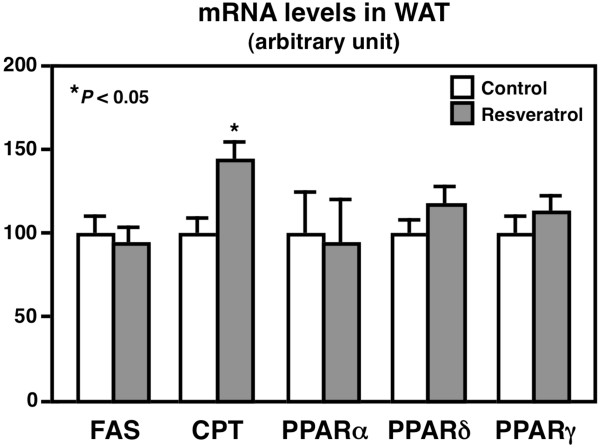
**The abundance of mRNAs related to lipid metabolism in white adipose tissue of OLETF rats.** The rats were fed a control diet or the resveratrol diet for 4 weeks. The values are expressed as the mean ± standard error for six mice. See Table 1 for the composition of the diets. ^*^ Significant (*P* < 0.05) difference between the control and resveratrol groups of OLETF rats. CPT, carnitine palmitoyltransferase; FAS, fatty acid synthase; PPAR, peroxisome proliferator-activated receptor.

To gain insight into the effect of dietary resveratrol on lipid metabolism in adipose tissue, we analyzed the mRNA expression of lipid metabolism-related genes by real-time RT-PCR. Previous studies have reported that body fat-lowering effect of resveratrol is mediated by down-regulation of adipogenesis in high-fat-fed mice and rats [14,15]. In contrast, the mRNA levels of FAS, a key enzyme in fatty acid synthesis, were not altered by dietary resveratrol in non-high-fat diet-fed OLETF rats (Figure 4). These results suggest that resveratrol is likely to modulate lipogenesis in a dietary fat-dependent manner. On the other hand, the mRNA levels of CPT, a key enzyme in fatty acid beta-oxidation, were significantly increased in the perirenal WAT of resveratrol-fed OLETF rats (Figure 4). Although the mRNA levels of PPARs, nuclear receptors related to the regulation of lipolysis and adipocyte differentiation, were not altered in this study (Figure 4), previous reports indicated that resveratrol regulates gene expression as a PPARα agonist *in vitro*[31,32]. Therefore, dietary resveratrol may enhance lipolytic gene expression through the activation of PPARα as a ligand in obese rats. Thus, we propose that the increase in CPT mRNA expression (possibly through the activation of PPARα) might contribute to the partial enhancement of fat oxidation by dietary resveratrol in non-high-fat diet-fed OLETF rats.

## Conclusion

In conclusion, our study showed that resveratrol has the potential to suppress body fat accumulation in non-high-fat diet-fed obese OLETF rats. The effect was, at least in part, attributable to the enhancement of fat oxidation and sparing actions for carbohydrates and protein in resveratrol-fed OLETF rats. Further studies are necessary to evaluate the effect of dietary resveratrol on nutrient metabolism in several animal models and determine the lowest effective concentration.

## Abbreviations

CPT: Carnitine palmitoyltransferase; FAS: Fatty acid synthase; OLETF: Otsuka Long-Evans Tokushima fatty; PPAR: Peroxisome proliferator-activated receptor; RQ: Respiratory quotient; UN: Urinary nitrogen; WAT: White adipose tissue.

## Competing interests

The authors declare that they have no competing interests.

## Authors’ contributions

KN made substantial contributions to the conception and design of the study, performing the experiment, assembling, analyzing and interpreting the data and drafting the manuscript. TJ and SK participated in the experimental work and in collecting, assembling, analyzing the data. TY contributed to planning the experiment and discussing the results. All authors read and approved the final manuscript.
